# Does impaired O_2_ delivery during exercise accentuate central and peripheral fatigue in patients with coexistent COPD-CHF?

**DOI:** 10.3389/fphys.2014.00514

**Published:** 2015-01-07

**Authors:** Mayron F. Oliveira, Joel T. J. Zelt, Joshua H. Jones, Daniel M. Hirai, Denis E. O'Donnell, Samuel Verges, J. Alberto Neder

**Affiliations:** ^1^Pulmonary Function and Clinical Exercise Physiology Unit (SEFICE), Respiratory Division, Department of Medicine, School of Medicine, Federal University of São Paulo (UNIFESP)São Paulo, Brazil; ^2^Laboratory of Clinical Exercise Physiology, Division of Respiratory and Critical Care Medicine, Department of Medicine, Queen's UniversityKingston, ON, Canada; ^3^Respiratory Investigation Unit, Division of Respiratory and Critical Care Medicine, Department of Medicine, Queen's UniversityKingston, ON, Canada; ^4^HP2 Laboratory, Grenoble Alpes UniversityGrenoble, France

**Keywords:** chronic heart failure, chronic obstructive pulmonary disease, oxygenation, respiratory muscle, skeletal muscle

## Abstract

Impairment in oxygen (O_2_) delivery to the central nervous system (“brain”) and skeletal locomotor muscle during exercise has been associated with central and peripheral neuromuscular fatigue in healthy humans. From a clinical perspective, impaired tissue O_2_ transport is a key pathophysiological mechanism shared by cardiopulmonary diseases, such as chronic obstructive pulmonary disease (COPD) and chronic heart failure (CHF). In addition to arterial hypoxemic conditions in COPD, there is growing evidence that cerebral and muscle blood flow and oxygenation can be reduced during exercise in both isolated COPD and CHF. Compromised cardiac output due to impaired cardiopulmonary function/interactions and blood flow redistribution to the overloaded respiratory muscles (i.e., ↑work of breathing) may underpin these abnormalities. Unfortunately, COPD and CHF coexist in almost a third of elderly patients making these mechanisms potentially more relevant to exercise intolerance. In this context, it remains unknown whether decreased O_2_ delivery accentuates neuromuscular manifestations of central and peripheral fatigue in coexistent COPD-CHF. If this holds true, it is conceivable that delivering a low-density gas mixture (heliox) through non-invasive positive pressure ventilation could ameliorate cardiopulmonary function/interactions and reduce the work of breathing during exercise in these patients. The major consequence would be increased O_2_ delivery to the brain and active muscles with potential benefits to exercise capacity (i.e., ↓central and peripheral neuromuscular fatigue, respectively). We therefore hypothesize that patients with coexistent COPD-CHF stop exercising prematurely due to impaired central motor drive and muscle contractility as the cardiorespiratory system fails to deliver sufficient O_2_ to simultaneously attend the metabolic demands of the brain and the active limb muscles.

## Introduction

The ability to sustain daily physical activities is inextricably linked to the integrity of the O_2_ transport pathway, which in turn depends on several multi-organ interactions involving the heart and vessels, lungs, respiratory/peripheral muscles and voluntary and autonomic nervous systems. Impaired tissue O_2_ transport due to altered perfusive and/or diffusive mechanisms are key pathophysiological abnormalities shared by two common cardiopulmonary diseases: chronic obstructive pulmonary disease (COPD) and chronic heart failure with reduced left ventricular ejection fraction (CHF). Unfortunately, COPD and CHF coexist in approximately 1 out of 3 elderly patients with a primary diagnosis of either disease (Rutten et al., 2005). Despite being a leading cause of disability and mortality worldwide (Boudestein et al., 2009), the pathophysiological consequences of the COPD-CHF overlap have been grossly overlooked in the medical literature.

Respiratory-mechanical (e.g., lung hyperinflation and increased intra-thoracic pressures) and gas-exchange (hypoxemia) abnormalities are known to impair the cardiac output ($\dot{Q}$t) response (Chiappa et al., 2008) and its distribution (Borghi-Silva et al., 2008a) during exercise in advanced COPD. On the other hand, impaired lung perfusion and interstitial edema decrease lung diffusing capacity (Guazzi et al., 2005) and increase the work of breathing and thus respiratory muscle metabolic demand (Narkiewicz et al., 1999; Ponikowski et al., 2001) in patients with CHF. Resultant impairments in peripheral muscle O_2_ delivery increase the respiratory drive through afferent stimulation from intra-muscle type III and IV receptors, which further raises the ventilatory requirements to exertion in both COPD (Gagnon et al., 2012) and CHF (Piepoli et al., 1996). Moreover, fatiguing contractions of the diaphragm in both diseases might elicit a sympathetic-mediated metaboreflex and further reduce limb muscle blood flow to the working peripheral muscles (Amann, 2011; Dempsey et al., 2006). Altogether, these abnormalities are expected to be additive in patients with COPD-CHF overlap and may lead to critical reductions in O_2_ delivery (i.e., “hypoxic” and “ischemic” hypoxia) under the stress of physical activities. The consequences of derangements in O_2_ delivery are particularly relevant to tissues requiring a fine-tune balance between O_2_ delivery and utilization during exercise, such as the central nervous system (CNS) and the active skeletal muscles. Despite these previous considerations, however, it remains unknown whether in patients with COPD-CHF overlap (1) decreased O_2_ delivery leads to deficits in cerebral and active limb muscle oxygenation and, if this shows to be the case, (2) whether those deficits accentuate neuromuscular manifestations of central and peripheral fatigue, respectively.

## Peripheral and central neuromuscular fatigue

Muscle fatigue can be defined as a reversible reduction in the maximal voluntary force or power of a single muscle or muscle group whether or not the task can be sustained (Gandevia, 2001). Neuromuscular fatigue can originate at different levels of the motor pathway and is usually divided into central and peripheral components. *Peripheral fatigue* is produced by phenomena at or distal to the neuromuscular junction. It can be demonstrated by a reduction in force evoked by peripheral supramaximal nerve stimulation in the relaxed potentiated muscle. In some cases, mechanisms related to peripheral fatigue may not explain the entire fatigue-related decrease in maximal voluntary force and some fatigue can arise from modifications within the CNS (Millet and Lepers, 2004). *Central fatigue* is defined as a progressive failure to voluntarily activate the muscle which can originate at the spinal and/or supraspinal levels. Interestingly, the peripheral and central components of muscle fatigue are intrinsically related given that recruitment of peripheral motoneurons depends on descending drive from supraspinal sites, whereas central drive is modulated by a combination of excitatory and inhibitory reflex inputs from peripheral muscles, joints, tendons, and cutaneous afferents (Millet et al., 2011).

Peripheral changes can be investigated by stimulating the muscle in the relaxed state before, during and after the fatiguing exercise. A single evoked stimulus allows measurements of mechanical (twitch) and electromyographic (M-wave) responses. In order to fully activate the muscles under scrutiny, it is crucial to use a stimulation (electrical or magnetic) of supramaximal intensity. Of note, forceful voluntary contractions before stimulation are required to avoid the potential bias of exercise-induced potentiation when assessing fatigue (Millet et al., 2011). Different types of stimuli can be evoked to noninvasively investigate (i) neuromuscular propagation of action potentials along the sarcolemma (M-wave, high-frequency fatigue), (ii) excitation–contraction coupling (low-frequency fatigue; LFF) and (iii) intrinsic force (high-frequency stimulation at supramaximal intensity). LFF is the most common type of peripheral fatigue associated with reductions in muscle O_2_ delivery (Amann et al., 2006a; Katayama et al., 2007) and is characterized by slow recovery associated with impairments in the excitation–contraction coupling. LFF is particularly deleterious to patients' tolerance to exertion as it might last for many hours after induction (Edwards et al., 1977), thus limiting the capacity to perform repetitive tasks.

Maximal voluntary activation (VA), as estimated by twitch interpolation (Merton, 1954), is the most conventional approach to assess central fatigue occurring during exercise. This maneuver consists of a supramaximal stimulation of a peripheral nerve to artificially generate action potentials during a maximal voluntary contraction. If lower motoneurons are not fully recruited during the maximal voluntary contraction, or are not firing fast enough, then the evoked stimulus will generate additional force, termed superimposed twitch. The ratio between the superimposed twitch and a potentiated twitch elicited in the relaxed muscle allows the quantification of VA. A decrease in VA during or after sustained contractions suggests reduced motor drive that originates at or above the stimulation site on the axons of the lower motoneurons (i.e., at the spinal and/or supraspinal levels; central fatigue). Transcranial magnetic stimulation (TMS) is an alternative, non-invasive method to assess neuromuscular function by stimulating directly the motor cortex. It has been shown that VA can also be examined with TMS, thus allowing the description of the supraspinal component of central fatigue (Todd et al., 2003).

## Effects of O_2_ delivery on peripheral and central fatigue in health

Changes in O_2_ delivery may affect muscular performance and the rate of development of both central and peripheral fatigue (as reviewed by Amann and Calbet, 2008). Blunted O_2_ delivery during exercise has been shown to accentuate, while augmented O_2_ delivery has been demonstrated to reduce, the rate of development of peripheral fatigue during isolated as well as whole-body exercise in healthy subjects. For instance, measurement of quadriceps twitch force evoked by magnetic femoral nerve stimulation before and after intermittent submaximal isometric contractions under normoxic and hypoxic conditions showed greater peripheral fatigability in hypoxia (Katayama et al., 2007). A cycling bout of similar absolute workload and duration also induced greater impairment of quadriceps contractility in hypoxia compared to normoxia (Amann et al., 2006a). Accelerated development of peripheral fatigue under hypoxic conditions has also been shown for both the inspiratory and expiratory muscles using cervical and thoracic magnetic stimulation before and after isocapnic hyperpnea (Verges et al., 2010). Changes in intracellular metabolism and the rate of accumulation of metabolites related to impairments in excitation-contraction coupling (e.g., H^+^, inorganic phosphates) are thought to underlie the rapid development of peripheral muscle fatigue with reduced O_2_ delivery (Amann and Calbet, 2008).

The exacerbation of central fatigue with reduced cerebral O_2_ delivery during whole-body exercise in healthy subjects has been recently demonstrated by Goodall et al. (2012). Endurance-trained cyclists exercised at ~ 80% of their normoxic maximum power in both normoxia and hypoxia conditions. Exercise time to exhaustion was halved in hypoxia versus normoxia. Post-exercise peripheral fatigue assessed by femoral nerve stimulation did not differ while supraspinal fatigue assessed by transcranial stimulation was larger in hypoxia compared to normoxia. Furthermore, the post-exercise VA and the level of cerebral oxygen delivery during exercise were positively correlated, suggesting that increased central fatigue during whole-body hypoxic exercise resulted from impaired cerebral O_2_ availability.

It has also been proposed that sensory afferent feedback from the fatiguing muscles to the CNS may be a key modulator of central motor drive and, therefore, exercise performance. This suggests a link between peripheral (i.e., biochemical changes within the active muscle) and central (i.e., reductions in central motor drive to the working muscle) fatigue (Amann and Dempsey, 2008; Amann, 2011). In this sense, the magnitude of the inhibitory neural feedback is proportional to the rate of development of peripheral locomotor muscle fatigue. Given that the latter is highly sensitive to muscle O_2_ delivery, an increased rate of peripheral fatigue development during exercise in hypoxia would act as a dose-dependent trigger of central fatigue. This is supported by the similar levels of peripheral quadriceps fatigue observed after cycling time trials performed with various arterial oxygenation levels in healthy subjects (Amann et al., 2006b). This mechanism may, however, not apply entirely to conditions of severe hypoxia which lead to critically low levels of brain oxygenation. Under these circumstances, the direct effect of severe hypoxia on the brain and central motor drive may predominate over the inhibitory effects of somatosensory afferent feedback (Verges et al., 2012). Therefore, moderate hypoxia may limit exercise performance mainly by accelerating the rate of peripheral fatigue development, while severe hypoxia may limit exercise tolerance primarily due to impaired cerebral oxygenation and subsequent central fatigue.

## Respiratory-locomotor muscle interactions and O_2_ delivery in health

Hyperventilation during heavy sustained exercise (e.g., 85% maximal oxygen consumption, $\dot{V}$O_2_max) causes substantial increases in respiratory muscle work, possibly leading to diaphragm and expiratory muscle fatigue (Dempsey et al., 2006). Accumulation of metabolites in these muscles stimulates group IV phrenic afferents, which in turn can increase sympathetic vasoconstrictor activity in the active locomotor limbs via a supraspinal reflex (St Croix et al., 2000). In this context, a competitive relationship between respiratory and active locomotor muscle for limited $\dot{Q}$t ensues and possibly results in redistribution of blood flow (i.e., ↑blood flow to respiratory muscles a ↓blood flow to active locomotor muscles) (Harms et al., 1997).

The competition for blood flow between respiratory and active lower limb muscles and the development of peripheral locomotor muscle fatigue independent of changes in CaO_2_ have been investigated in healthy subjects during high intensity cycling exercise at similar work rates, duration of exercise, and CaO_2_ levels (Harms et al., 1997; Romer et al., 2006; Amann et al., 2007). When an exercise trial was performed with a mechanical ventilator (thus reducing inspiratory muscle pressure output by up to 70%), active limb blood flow was increased by ≥5% and leg $\dot{V}$O_2_ by 3% compared with the control condition without ventilatory assistance (Harms et al., 1997). Moreover, exercise-induced peripheral quadriceps fatigue (assessed by femoral nerve stimulation) was reduced by 30% with the mechanical ventilator compared with control when both trials were performed in normoxia (Romer et al., 2006), and by over 35% when both trials were performed in acute hypoxia (Amann et al., 2007). In contrast, enhancing inspiratory muscle work by about 80% with inspiratory resistive loading reduced active limb blood flow by over 10% compared with control, which approximately doubled the amount of post-exercise quadriceps muscle fatigue (Romer et al., 2006). It should be noted that, in healthy subjects, changes in limb blood flow induced by modifying the work of breathing and the subsequent effect on peripheral fatigue have been observed at exercise intensities greater than 80% of $\dot{V}$O_2_max (Amann et al., 2007). This may derive from the fact that, at those intensities, exhaustive exercise is capable of eliciting diaphragm fatigue and triggering the metaboreflex as described above (Dempsey et al., 2006). These findings thus suggest that, under hypoxemic conditions, both the decrease in CaO_2_ and the increase in respiratory muscle work (due to increased ventilation) might independently contribute to the development of fatigue during exercise.

## Lung-heart interactions and exercise O_2_ delivery in COPD

Expiratory flow limitation and lung hyperinflation that are only partially reversible to bronchodilator therapy are pathophysiological hallmarks of COPD (Langer et al., 2014). When expiratory flow-limitation is present during resting spontaneous breathing, end-expiratory lung volume is dynamically determined by and varies with the mechanical time constant for emptying (the product of resistance and compliance) of the respiratory system (O'Donnell et al., 2001). As the expiratory time declines with higher breathing frequency during exercise, a substantial amount of inhaled air is further “trapped” in the lung—a process termed dynamic hyperinflation (Stubbing et al., 1980; Johnson et al., 1999; O'Donnell et al., 2001; Calverley, 2006). Breathing in relatively high lung volumes during exercise is energetically and mechanically disadvantageous as dynamic compliance decreases and the respiratory muscles become less effective in generating the required pressures (O'Donnell et al., 2014). Consequently, the combination of increased ventilatory requirements (mainly secondary to increased ventilation/perfusion mismatching and/or hypoxemia) and abnormal dynamic ventilatory mechanics stress the already diminished cardiopulmonary reserves during exercise.

**Figure 1:**
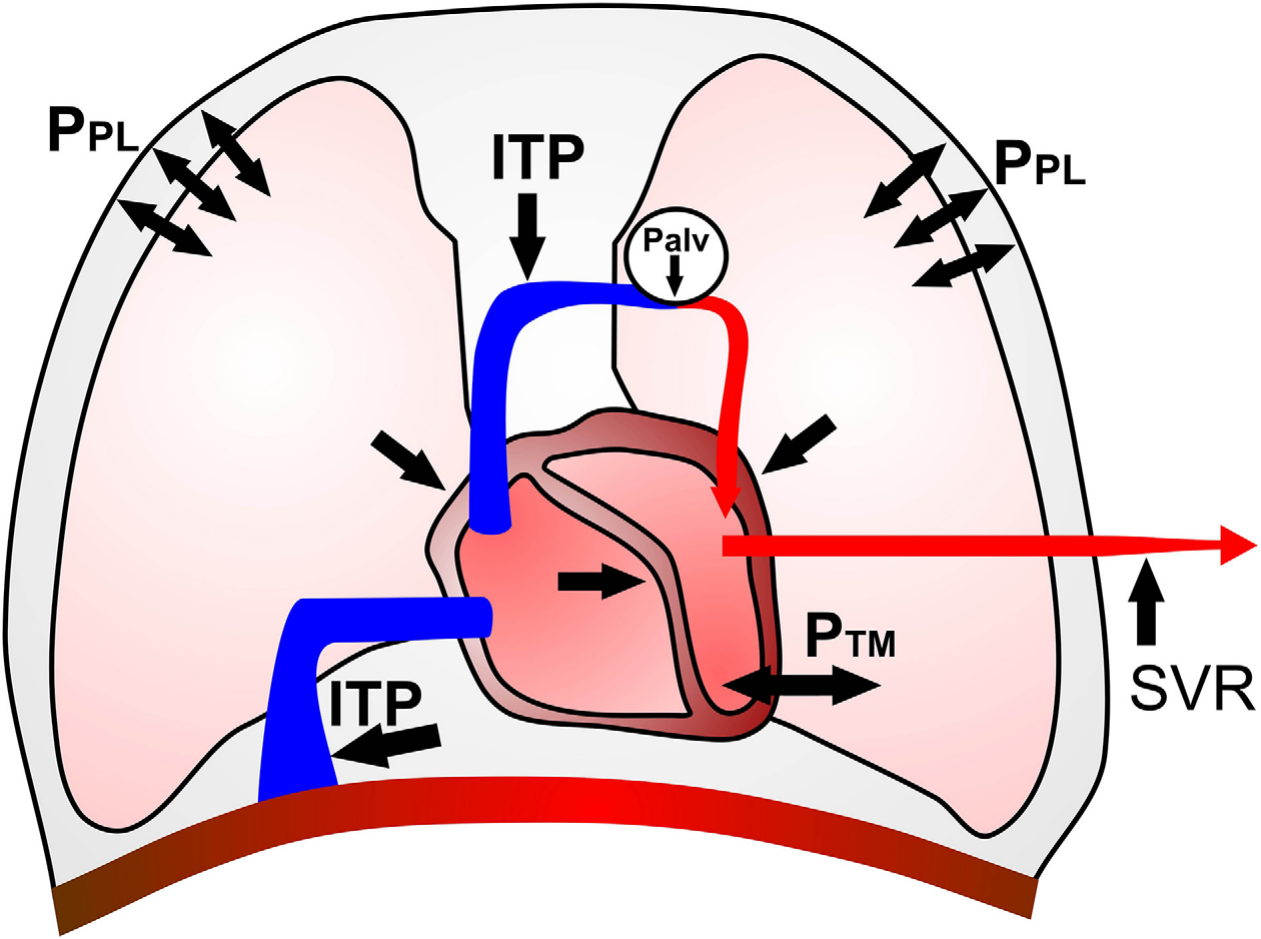
**Schematic representation of potential negative cardiopulmonary interactions in moderate to severe COPD**. Increases in mean intra-thoracic pressure (ITP) and large swings in pleural pressure (P_PL_) may reduce venous return and right ventricular (RV) preload. High P_PL_ swings, compression of juxta-alveolar capillaries and hypoxic vasoconstriction increase RV afterload and intra-cavitary pressures. The latter occurrence, in association with extrinsic compression of the right heart by the overdistended lungs, can impair RV relaxation and displace the inter-ventricular septum to the left. Reduced left heart filling pressures and dimensions may contribute to further impairments in stroke volume (SV). Large P_PL_ swings can also increase left ventricular (LV) afterload secondary to high transmural pressures (P_TM_). Moreover, decreased aortic impedance and augmented systemic vascular resistance (SVR) further increase LV afterload, thus compromising cardiac output ($\dot{Q}$t). Of note, the relative contribution of each of the above factors is likely to vary according to different phases of respiratory and cardiac cycles.

There is also evidence that the mechanical interactions between the cardiac and ventilatory pumps negatively affect the cardiovascular responses to exercise in COPD (Figure 1). Generation of relatively more negative intrathoracic pressures during forceful inspiration to overcome the intrinsic positive end-expiratory pressure act to increase left ventricular transmural pressure and afterload (Ranieri et al., 1996). Forceful expiratory muscle recruitment in the presence of expiratory flow limitation may fail to produce additional flow but might increase markedly the alveolar pressures. The latter act directly on the blood vessels surrounding the alveolar walls, leading to mechanical compression and increasing vascular pressures above the venous pressures responsible for a normal venous return (Cassidy and Mitchell, 1981; Scharf, 1991). In addition, as pulmonary vascular resistance and pulmonary artery pressures rise, right ventricular ejection fraction fails to increase despite progressively higher right ventricular end-diastolic volume. In these conditions, left ventricular end-diastolic, end-systolic and stroke volumes may diminish because of a reduction in right ventricular ejection fraction and/or competition for space between the two sides of the heart within the pericardium (i.e., ventricular interdependence) (Potter et al., 1971; Robotham et al., 1978; Nóbrega et al., 1994; Saito et al., 1999; Stark-Leyva et al., 2004; Aliverti et al., 2005; Miller et al., 2005, 2006). This has important clinical implications given that the rate at which $\dot{Q}$t increases from rest to exercise is a strong determinant of peripheral O_2_ delivery in patients with COPD free of heart disease (Chiappa et al., 2008).

Also interestingly, cerebral oxygenation has been found to be impaired during exercise in COPD patients even in the absence of hypoxemia (Oliveira et al., 2012). Strategies that were able to reduce the work of breathing and increase $\dot{Q}$t (e.g., non-invasive positive pressure ventilation) can improve cardiopulmonary function/interactions during exercise and ameliorate both muscle and cerebral oxygenation profiles in these patients (Borghi-Silva et al., 2008a; Rodrigues et al., 2013). Altogether, these data indicate that not only $\dot{Q}$t dynamics can be slowed from rest to exercise but also that its adequate distribution to key organs is disturbed—even in COPD patients with no evidence of CHF (Borghi-Silva et al., 2008a; Chiappa et al., 2008). This opens the perspective that these deleterious phenomena may be potentiated by the presence of concomitant CHF.

## Heart-lung interactions and exercise O_2_ delivery in CHF

It is generally accepted that reduced physical capacity with CHF is a direct effect of the cardiovascular and neurohumoral derangements that characterize the disease. Structural and functional cardiovascular impairments affect the O_2_ transport pathway and compromise the matching between O_2_ delivery and utilization particularly during exercise (Poole et al., 2012). As reviewed by Poole et al. (2012), in the presence of a failing heart, $\dot{Q}$t particularly during exercise is reduced consequent to a diminished ejection fraction, lower stroke volume (SV) and a heart rate (HR) response that is insufficient to fully compensate for the reduced SV. In an attempt to avoid further reductions in $\dot{Q}$t (and systemic blood pressure), there is a systemic sympathetically-mediated vasoconstriction which, when maintained over time, impairs the ability to distribute the reduced $\dot{Q}$t to and within skeletal muscle(s). Increased circulating levels of humoral mediators such as angiotensin, vasopressin, norepinephrine and endothelin-1 contribute to global vasoconstriction. Additionally, vasodilatory mechanisms are impaired not only due to intrinsic vascular abnormalities (Poole et al., 2012) but also secondary to intravascular sodium and water retention. Tissue O_2_ delivery might also be worsened further if CHF is accompanied by anemia due to dysfunctional iron metabolism and, possibly, marked inflammation. In addition, within skeletal muscles the capacity to utilize O_2_ can be impaired in more severe patients due to reductions in mitochondrial oxidative enzyme activity and volume density as well as mitochondrial dysfunction (Hirai et al., 1995; Belardinelli et al., 1997; Kindig et al., 1999; Diederich et al., 2002; Copp et al., 2010; Bowen et al., 2012; Poole et al., 2012).

There is also evidence that CHF patients might develop pulmonary dysfunction including ventilation-perfusion ($\dot{V}$/$\dot{Q}$) mismatch (Agostoni et al., 2006), reduced O_2_ diffusing capacity (Guazzi et al., 2005) and diminished respiratory muscle strength and endurance (Agostoni et al., 2002). While $\dot{V}$/$\dot{Q}$ mismatch and diffusive deficiencies may not always lead to arterial hypoxemia in CHF, restrictive and obstructive abnormalities combined with the sensitization of peripheral chemoreceptors (carotid bodies) result in increased work of breathing (Narkiewicz et al., 1999; Ponikowski et al., 2001). In addition, non-smokers with CHF can also present with some degree of dynamic hyperinflation during exercise, probably due to increased collapsibility of the small airways (Ribeiro et al., 2009). It is therefore conceivable that the concomitance of COPD could potentiate these respiratory abnormalities in patients with CHF.

## Respiratory-locomotor muscle interactions and O_2_ delivery in COPD and CHF during exercise

Experimental evidence suggests that a similar competitive relationship between respiratory and active locomotor muscles for limited $\dot{Q}$t also exists in patients with COPD or CHF during exercise. For instance, enhancement in the cardiorespiratory adjustments to exercise through less dynamic hyperinflation (e.g., via bronchodilatation, Berton et al., 2010, non-invasive positive pressure ventilation, Borghi-Silva et al., 2008a, and low density gas breathing, Chiappa et al., 2009) accelerates active lower limb muscle O_2_ delivery during metabolic transitions in patients with moderate to severe COPD. Moreover, unloading of the respiratory muscles via proportional assist ventilation improves peripheral contracting muscle oxygenation in COPD patients even if $\dot{Q}$t and CaO_2_ remain unaltered (Borghi-Silva et al., 2008a). In line with these findings, Amann et al. (2010) evaluated the effect of various interventions improving arterial oxygenation and/or reducing the work of breathing during cycling (via proportional assist ventilation, heliox and hyperoxia) on quadriceps muscle fatigue (assessed by femoral nerve stimulation) in COPD patients. These interventions attenuated exercise-induced quadriceps fatigue by one third compared to the control condition. Collectively, these data suggest that the high susceptibility to locomotor muscle fatigue in patients with COPD (Mador et al., 2003, 2005) is, at least in part, attributable to arterial hypoxemia and/or to excessive respiratory muscle work.

Similar findings have been reported in patients with CHF in isolation: blood flow to active skeletal muscle can be reduced not only due to lowered $\dot{Q}$t and heightened sympathetic vasoconstriction but also via a “stealing” mechanism triggered by enhanced work of breathing during exercise (Harms et al., 1997; Sheel et al., 2001; Iandelli et al., 2002; Dempsey et al., 2006). In fact, respiratory muscle unloading (via proportional assist ventilation) has been demonstrated to promote beneficial effects on the oxygenation status of the exercising muscles at similar $\dot{Q}$t and CaO_2_ in patients with advanced CHF (Borghi-Silva et al., 2008b). These data suggest that unloaded breathing during exercise may minimize the “stealing” effect and lead to increased blood flow to the active locomotor muscles of CHF patients. Nonetheless, given that exercise has not been performed to the limit of tolerance nor has muscle fatigue been assessed in previous studies, it is not possible to determine the potential contribution of this blood flow “stealing” mechanism to the exercise capacity of these patients.

## Hypothesis

**Figure 2:**
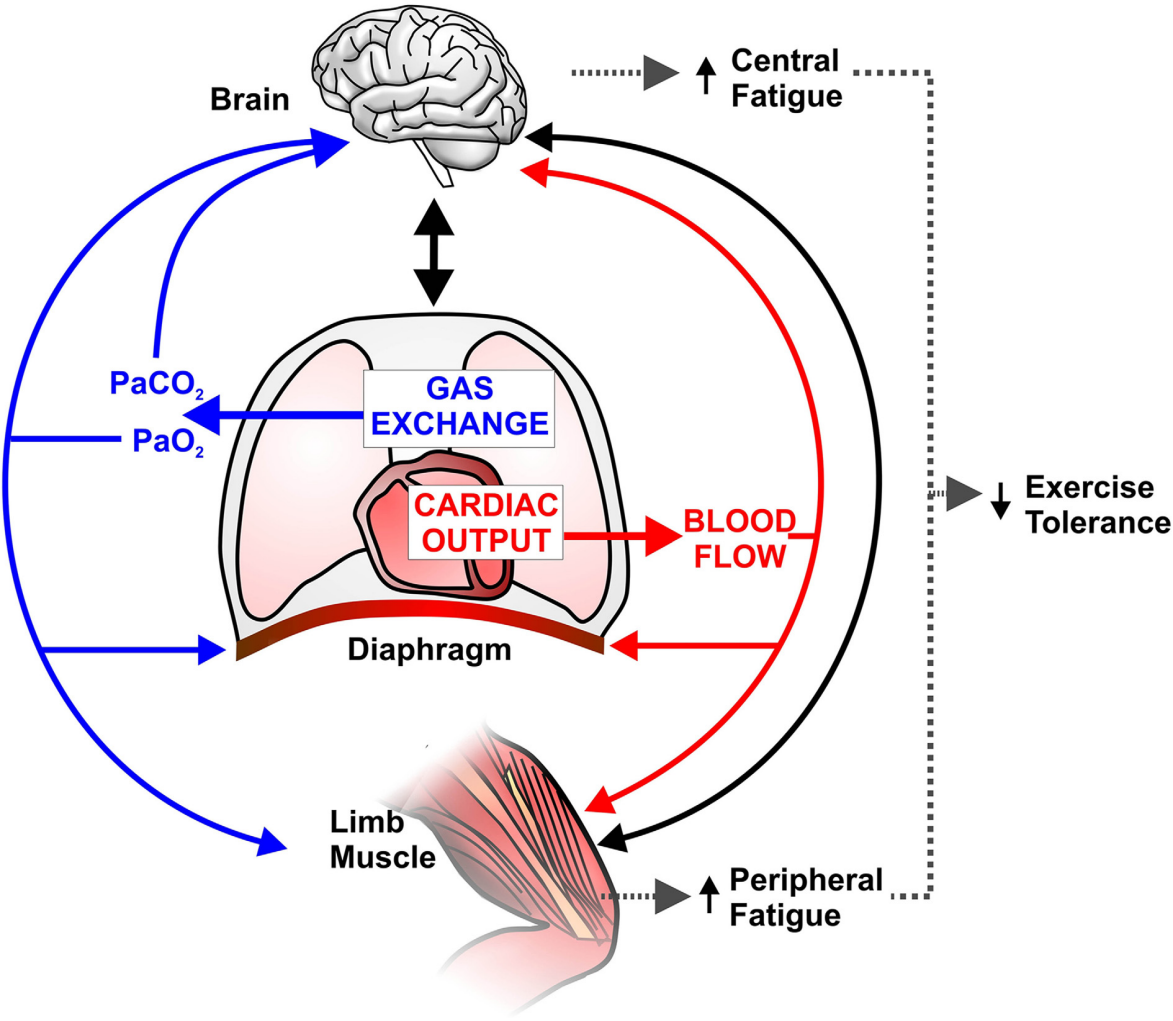
**Schematic representation of potential implications of abnormal pulmonary gas exchange and central hemodynamics on central nervous system (brain) and peripheral skeletal muscle function during exercise in combined COPD-CHF**. Compromised O_2_ delivery to brain and active limb muscles can occur as a consequence of impairments in gas exchange (e.g., ↓arterial O_2_ pressure; PaO_2_) and/or decreased cardiac output ($\dot{Q}$t) and thus blood flow. A large fraction of an already reduced $\dot{Q}$t can be directed to the overloaded respiratory muscles (due to ↑work of breathing), therefore further decreasing active limb muscle perfusion and accentuating peripheral fatigue. *Solid black lines* indicate that increased afferent information from the respiratory/peripheral muscles and/or impaired cerebral oxygenation may decrease motor drive (i.e., central fatigue). In this context, it is conceivable that central and peripheral fatigue potentiate each other and contribute to early exercise cessation in coexistent COPD-CHF.

Based on the body of evidence presented above, it is hypothesized that impaired O_2_ delivery to both the pre-frontal cortex and active limb muscles in coexistent COPD-CHF contributes significantly to reduced central motor drive and accelerated peripheral muscle fatigue development, respectively (Figure 2).

## Evaluation of the hypothesis

We propose to test our hypothesis by investigating the neuromuscular consequences (both at the central and peripheral levels) of improved cerebral and active limb skeletal muscle O_2_ delivery during cycling exercise in patients with coexistent COPD-CHF. We firstly expect that central and peripheral fatigue would be related to impairments in cerebral and peripheral exercising muscle oxygenation, respectively, in the control condition. We then anticipate that these abnormalities would be simultaneously alleviated by a strategy aimed at improving cardiopulmonary function/interactions and reducing the work of breathing (via non-invasive positive pressure ventilation; NIPPV). Utilization of this intervention in combined COPD-CHF patients is expected to slow the rate of central and peripheral fatigue development and enhance physical capacity. We envisage that further improvements in cardiopulmonary function/interactions and exercise tolerance of COPD-CHF patients would be reached by adding a low-density gas mixture (e.g., heliox to decrease airflow resistance) to NIPPV. We also reason that further increases in cerebral and skeletal muscle O_2_ delivery would be induced by progressively increasing inspired O_2_ fraction in different hyperoxic heliox gas mixtures. This would allow us to interrogate individual contributions of each of these interventions on systemic O_2_ delivery.

Standard cardiopulmonary responses, $\dot{Q}$t and exertional ratings (leg fatigue and dyspnea) will be continuously monitored during the transition from rest to exercise. By placing gastric and esophageal pressure catheters, inspiratory and expiratory muscle force production will be assessed together with the work of breathing. Respiratory and locomotor muscle activity will be evaluated by surface electromyography. Patients will cycle with near-infrared spectroscopy (NIRS) probes placed on the pre-frontal cortex and the quadriceps muscle. An optically-dense dye (indocyanine green) will be intermittently injected in the venous circulation and its rate of appearance will be determined by NIRS. This will allow the evaluation of tissue blood flow and microvascular oxygenation (i.e., index of O_2_ delivery-to-utilization (mismatching). Arterial blood gases will be assessed at rest and during exercise. Neuromuscular function via femoral and phrenic nerve magnetic stimulation will be evaluated before and immediately after exercise to investigate changes in evoked electromyographic and mechanical responses of the quadriceps and respiratory muscles.

## Medical consequences of the hypothesis

Despite mounting evidence that COPD-CHF overlap represents a growing cause of physical incapacity in the western world (Rutten et al., 2005; Boudestein et al., 2009), little is known about the underlying mechanisms. This important gap in current knowledge needs to be addressed to facilitate the development of novel and effective therapeutic and rehabilitative strategies tailored to the specific needs of these patients. Confirmation of the study hypothesis could open the perspective that enhancing O_2_ delivery and/or decreasing O_2_ requirements within the brain and active limb skeletal muscle positively impact upon tolerance to exertion in patients with combined COPD-CHF. This might set the scene for randomized controlled trials with pharmacological (e.g., antioxidant and anti-inflammatory agents), non-pharmacological (e.g., non-invasive ventilation, normoxic/hyperoxic heliox) and dietary (e.g., oral nitrate supplementation) interventions that might delay central and peripheral neuromuscular fatigue development in this patient population.

## Conflict of interest statement

The authors declare that the research was conducted in the absence of any commercial or financial relationships that could be construed as a potential conflict of interest.

## Abbreviations

CaO_2_: arterial oxygen content
CHF: chronic heart failure
CNS: central nervous system
COPD: chronic obstructive pulmonary disease
HR: heart rate
ITP: intra-thoracic pressure
LFF: low-frequency fatigue
LV: left ventricle
NIPPV: non-invasive positive pressure ventilation
NIRS: near-infrared spectroscopy
PaCO_2_: arterial partial pressure of carbon dioxide
Palv: alveolar pressure
PaO_2_: arterial partial pressure of oxygen
PEEPi: intrinsic positive end-expiratory pressure
P_PL_: pleural pressure
P_TM_: transmural pressure
$\dot{Q}$t: cardiac output
RV: right ventricle
SV: stroke volume
SVR: systemic vascular resistance
TMS: transcranial magnetic stimulation
VA: maximal voluntary activation
$\dot{V}$O_2_: oxygen uptake
$\dot{V}$O_2_max: maximal oxygen uptake
$\dot{V}$/$\dot{Q}$: ventilation-perfusion relationship
